# Association between lipid profiles and viral respiratory infections in human sputum samples

**DOI:** 10.1186/s12931-022-02091-w

**Published:** 2022-07-02

**Authors:** Sara T. Humes, Nicole Iovine, Cindy Prins, Timothy J. Garrett, John A. Lednicky, Eric S. Coker, Tara Sabo-Attwood

**Affiliations:** 1 Department of Environmental and Global Health, Center for Environmental and Human Toxicology, Emerging Pathogens Institute, University of Florida, Gainesville, Florida 32611 USA; 2 Division of Infectious Diseases & Global Medicine, University of Florida, Gainesville, Florida 32611 USA; 3 Department of Epidemiology, University of Florida, Gainesville, Florida 32611 USA; 4 Department of Pathology, Immunology and Laboratory Medicine and Southeast Center for Integrated Metabolomics, University of Florida, Gainesville, Florida 32611 USA

**Keywords:** Lipidomics, Viral infection, Sputum, Influenza, Bayesian regression

## Abstract

**Background:**

Respiratory infections such as influenza account for significant global mortality each year. Generating lipid profiles is a novel and emerging research approach that may provide new insights regarding the development and progression of priority respiratory infections. We hypothesized that select clusters of lipids in human sputum would be associated with specific viral infections (Influenza (H1N1, H3N2) or Rhinovirus).

**Methods:**

Lipid identification and semi-quantitation was determined with liquid chromatography and high-resolution mass spectrometry in induced sputum from individuals with confirmed respiratory infections (influenza (H1N1, H3N2) or rhinovirus). Clusters of lipid species and associations between lipid profiles and the type of respiratory viral agent was determined using Bayesian profile regression and multinomial logistic regression.

**Results:**

More than 600 lipid compounds were identified across the sputum samples with the most abundant lipid classes identified as triglycerides (TG), phosphatidylethanolamines (PE), phosphatidylcholines (PC), Sphingomyelins (SM), ether-PC, and ether-PE. A total of 12 lipid species were significantly different when stratified by infection type and included acylcarnitine (AcCar) (10:1, 16:1, 18:2), diacylglycerols (DG) (16:0_18:0, 18:0_18:0), Lysophosphatidylcholine (LPC) (12:0, 20:5), PE (18:0_18:0), and TG (14:1_16:0_18:2, 15:0_17:0_19:0, 16:0_17:0_18:0, 19:0_19:0_19:0). Cluster analysis yielded three clusters of lipid profiles that were driven by just 10 lipid species (TGs and DGs). Cluster 1 had the highest levels of each lipid species and the highest prevalence of influenza A H3 infection (56%, n = 5) whereas cluster 3 had lower levels of each lipid species and the highest prevalence of rhinovirus (60%; n = 6). Using cluster 3 as the reference group, the crude odds of influenza A H3 infection compared to rhinovirus in cluster 1 was significantly (p = 0.047) higher (OR = 15.00 [95% CI: 1.03, 218.29]). After adjustment for confounders (smoking status and pulmonary comorbidities), the odds ratio (OR) became only marginally significant (p = 0.099), but the magnitude of the effect estimate was similar (OR = 16.00 [0.59, 433.03]).

**Conclusions:**

In this study, human sputum lipid profiles were shown to be associated with distinct types of viral infection. Better understanding the relationship between respiratory infections of global importance and lipids contributes to advancing knowledge of pathogenesis of infections including identifying populations with increased susceptibility and developing effective therapeutics and biomarkers of health status.

**Supplementary Information:**

The online version contains supplementary material available at 10.1186/s12931-022-02091-w.

## Background

Respiratory infections are a major public health issue and are among the top ten leading causes of death worldwide [1]. Just in the U.S., more than 500 million infections occur annually at a loss of 40 billion dollars and up to 100 million school and work days lost [2]. The Centers for Disease Control and Prevention (CDC) estimates that each year influenza virus infections account for up to 35.6 million cases of illness, 710,000 hospitalizations, and 56,000 deaths [3]. In response to the tremendous economic and health burden, the World Health Organization (WHO) has set a research agenda for influenza, which addresses the need to reduce the risk of pandemic emergence, limit spread, minimize disease burden, optimize treatment, and promote development of new public health tools [4]. To address these areas, it is imperative that we better understand individual factors that impact susceptibility to infection or severity of infection, and with that knowledge, develop ways to improve prognosis and therapeutic interventions.

A growing area of research is centered on the role of lipids in modulating host–pathogen interactions and the host immune response. The discovery that lipids play important roles in the context of lung infections is not new, although the vast number of lipids, particularly at the species level, have challenged our ability to define their specific roles. More recent technological advancements, such as mass spectrometry, have improved our ability to search for and identify lipid profiles and specific lipid classes or species that vary with  disease conditions. For example, data from recent ‘omic’ studies have emphasized possible links between lipids and the innate immune response to viral respiratory infections that specifically include viral sensing and modulation of immune signaling pathways [5–9] which both enhances the viral lifecycle and assists the host immune response [10].

Studies have provided evidence that lipids and their metabolites can be used to differentiate between disease states such as community acquired and non-community acquired pneumonia cases [13] and between healthy controls and asthmatic patients [14]. Few studies, however, have investigated a role for lipids in respiratory infections using sputum samples, and no studies have investigated associations between sputum lipid profiles and respiratory infections.

Sputum is routinely collected in a relatively noninvasive manner and has been shown to reflect the contents of BALF and epithelial lining fluid [12, 16]. Several studies have also demonstrated that sputum is as good or better than nasal swabs/aspirates for detection of influenza viruses, bacterial pneumonia, and other respiratory infections [17–19] that may colonize the lower airways.

Therefore, the objective of this study was to examine the associations between lipid profiles of sputum and the type of respiratory viral agent. We hypothesized that lipid profiles would cluster together based on specific causative viral agents of infection.

## Methods

### Sample collection and processing

The sputum samples for this study were collected from University of Florida (UF) Health Shands Hospital over a six-month period (January-June 2019), which included the typical peak of flu season defined by the CDC (https://www.cdc.gov/flu/about/season/flu-season.htm). Sputum samples were collected as part of routine care for diagnostic testing by the UF Health Shands Clinical Microbiology Laboratory. After collection, the samples were processed for requested tests and any leftover sample was eligible for inclusion in the study and stored at 4 °C.

Sputum samples were excluded if they contained > 10 squamous epithelial cells after gram stain and under bright-field microscopy (10X), defined as few or none (on a scale of none, few, moderate, many) as they had a high likelihood of saliva contamination. Because the sputum came from patients with moderate to severe respiratory infections the samples likely contained moderate to many (> 25) leukocytes. The study population was limited to patients for which a nasopharyngeal (NP) swab was also tested for pathogen identification using the BioFire FilmArray® system, a multiplex PCR capable of identifying 20 respiratory pathogens. Samples for this study were collected from children and adults irrespective of mechanical ventilation status, however those with suspected tuberculosis infection (acid-fast bacilli testing by Clinical Laboratory) and those who were pregnant or otherwise immunocompromised (if known at time of sample collection) were excluded.

Once an eligible sample was identified via surveillance of Clinical Laboratory records, it was obtained and given a unique identifier. The results from the BioFire FilmArray® tests of NP swabs in the Clinical Laboratory were used to determine the causative agent of infection for each patient. If there was sufficient sample volume (at least four mL), the sputum was tested using the BioFire FilmArray® 1.5 Classic Respiratory Panel to examine concordance with the same test but different sample type (sputum vs. NP swab, Additional file 1: Table S2). Since the BioFire FilmArray® 1.5 Classic Respiratory Panel is optimized for use with NP swab samples, we had to optimize preparation of sputum samples, which requires incubation with a mucolytic agent, in this case Dithiothreitol (DTT). Details of this experiment are described in the supplemental section (Additional file 1: Table S1) confirming that the DTT did not interfere with the BioFire assay. The remaining sputum was aliquoted and stored at -80 °C pending lipidomic analyses.

### Lipidomics

Once all the samples were collected, one aliquot of each sample was thawed and inactivated with methanol before being transferred to the Mass Spectrometry Core of the Southeast Center for Integrated Metabolomics (SECIM) for further extraction and analysis.

Samples were normalized to a sample protein concentration of 500 μg/mL, as measured by the Qubit™ Protein Assay Kit (ThermoFisher Scientific). Red Cross Plasma (RCP) was extracted with the samples as a control. Both the samples and RCP were spiked wtih 10 × dilution of lipid internal standard solution. An extraction blank (saline without internal standards) was also included. The lipids were extracted using a methanol-chloroform extraction, following the Folch method [20]. Pooled samples from each group (by infection type) were prepared by combining a small volume of each sample within a group. Mobile phase blank (2-propanol) and Neat QC (2:2:196 (v/v/v) lipid internal standards solution/ lipid injection standards solution/2-propanol) were also included.

Untargeted LC–MS lipidomics profiling was performed on a Thermo Q-Exactive Orbitrap mass spectrometer with Dionex UHPLC and autosampler (Thermo Scientific, San Jose, CA). All samples, blanks, and controls were analyzed in positive and negative heated electrospray ionization with a mass resolution of 35,000 at *m/z* 200 as separate injections. Separation was achieved on an Acquity BEH C18 1.7 µm, 50 × 2.1 mm, 1.7 µm column with mobile phase A as 60:40 Acetonitrile:Water containing 10 mM Ammonium formate with 0.1% formic acid and mobile phase B as 90:8:2 2-propanol: acetonitrile:water containing 10 mM ammonium formate with 0.1% formic acid. The flow rate was 500 µL/min with a column temperature of 50 °C. 5 µL was injected for negative ions and 3 µL for positive ions. The samples were randomized and analyzed following an injection sequence consisting of 3 mobile phase, Neat QC, RCP, extraction blank and 10 samples. Every 10 samples were bracketed by a mobile phase blank, Neat QC, RCP and extraction blank injections. Full Scan MS was acquired from individual and pooled samples in order to compare lipid intensities across groups. MS/MS spectra from data-dependent (ddMS2-top10) and all-ion fragmentation (AIF) were acquired on RCP, pooled samples, and/or representative samples from each group for lipid identification purposes. Only lipids that were identified by MS/MS were included in the statistical analyses due to the presence of polyethylene glycol (PEG) at the early elution times.

### Retrospective chart review

After all sputum samples were obtained and coded, retrospective chart review was conducted to abstract key patient characteristics. Electronic medical records were searched for the following information: (1) Demographics (gender, age range, body mass index (BMI), occupation); (2) Smoking status, current medications (specifically antibiotics, statins, steroids), travel history, flu vaccine status; (3) Comorbidities (cardiovascular disease, malignancy, immunodeficiency, pulmonary diseases such as asthma, COPD, recent upper respiratory infection, fibrosis); (4) Date of sample collection, microbiology lab results, chest x-ray results; (5) Any physician diagnosis coinciding with sputum collection; (6) Any pulmonary interventions performed prior to sputum collection (such as mechanical ventilation, nebulizer treatment, supportive oxygen).

### Statistical analyses

Data from positive and negative ion modes were analyzed separately using in-house LipidMatch Flow software. LipidMatch Flow was used for file conversion (MSConvert, Proteowizard), peak picking (MzMine 2.26), blank feature filtering, and identification [21]. Data analysis was performed on the three sample groups (stratified by infection type—Influenza A H1-2009, Influenza A H3, and Rhinovirus) using MetaboAnalyst 3.0. We utilized LION, a lipid ontology enrichment web-based tool (http://www.lipidontology.com/) to generate information on pathways that our lists of lipids enriched for using both target list modes for all lipids and ranking mode for comparisons between infection types.

Further analysis using Bayesian Profile Regression method [22, 23] was utilized to first identify clusters of individuals with similar patterns of lipid expression (i.e. similar lipid phenotypes). In our implementation of Bayesian profile regression, we leveraged a novel variable selection method in order to identify which lipid species were driving the observed clustering pattern. Prior to Bayesian profile regression, we first screened for candidate lipid species using the Kruskal–Wallis statistical test to determine if lipid species levels varied significantly by the type of respiratory viral infection. Given the limitations of the small sample size in our study, adjustment of p-values is overly conservative. We therefore applied a variable selection cut-off whereby those lipid species with a p-value ≤ 0.2 from the Kruskal–Wallis test were included for further clustering analyses. We emphasize that this step is for screening candidate lipid species for cluster analysis and that statistical inference in our study is based strictly on regression analysis (described below). For the cluster analysis with Bayesian profile regression, the peak height of each selected lipid species was first categorized into tertiles because of the highly skewed distributions of lipid quantities. We note that while Bayesian profile regression can be a type of supervised algorithm when including the outcome, in our study the clustering algorithm was unsupervised in that the outcome was not included in the model to inform the clustering allocation. After individual sputum samples were allocated to a cluster, we computed Z-scores of median peak height of the different lipid species in order to visualize with a heat map how the individuals’ levels of lipid species were jointly distributed within each cluster.

Finally, crude and adjusted multinomial logistic regression were performed to test the relationship between cluster and prevalence of type of infection. In these final models, the clusters were fit as three-level factor variables and the type of respiratory viral agent was set as the outcome (influenza A H1-2009, influenza A H3, rhinovirus [reference]). A causal diagram or directed acyclic graph (DAG; DAGitty version 2.3) was constructed to visualize and identify the confounding variables that may be influencing the relationship between lung lipids (exposure) and viral respiratory infection (outcome) (Additional file 1: Figure S1). Our review of the literature, in addition to our construction of a DAG, indicated that age, BMI, smoking status, and presence of pulmonary comorbidities such as asthma or COPD could act as possible confounders in our study. We then included only those covariates for adjustment in multinomial regression if they showed a significant association (p < 0.05) with infection type in bivariate analyses. We emphasize that one important benefit of basing inference in our study using a dimension reduction technique is that we avoid multiple tests of association, which would otherwise require adjustment of p-values that would be overly conservative in our study with such a small sample size.

## Results

### Subject characteristics

Thirty-five sputum samples were collected between January and June of 2019 for the study. Of those thirty-five samples, five were excluded from analysis in order to conduct more meaningful comparisons due to age (three total samples from children), pregnancy (one sample; patient’s pregnancy status identified after sample collection), and viral agent type (one patient positive for parainfluenza). The remaining thirty samples were used for lipidomic analyses and retrospective chart review. The study population consisted of 16 males and 14 females with 9 positives for influenza A H1-2009, 11 positives for influenza A H3, and 10 positives for rhinovirus. Other study population characteristics, such as age, body mass index (BMI), smoking status, and pulmonary comorbidities, are summarized in Table 1. Concordance of viral status between NP swab and sputum samples was performed on seven samples using the BioFire FilmArray® 1.5 Classic Respiratory Panel and results showed 100% agreement between sample types (Additional file 1: Table S2).

**Table 1:** Description of patient characteristics

Population characteristics	Number (%)
Total	30
*Sex*	
Male	16 (53.33)
Female	14 (46.67)
*Age*	
18-29	0 (0.00)
30-39	2 (6.67)
40-49	3 (10.00)
50-59	5 (16.67)
60-69	10 (33.33)
70-79	2 (6.67)
80-89	7 (23.33)
≥90	1 (3.33)
*BMI*	
Underweight (<18.5)	2 (6.67)
Normal (18.5 to <25)	9 (30.00)
Overweight (25 to <30)	11 (36.67)
Obese (≥30)	8 (26.67)
*Smoking Status*	
Never	11 (36.67)
Former	10 (33.33)
Current	9 (30.00)
*Pulmonary Comorbidities*	
None	17 (56.67)
Asthma or COPD	13 (43.33)
*Mechanical Ventilation*	
None	21 (70.00)
Required	9 (30.00)
*Infection Present*	
Influenza A H1-2009	9 (30.00)
Influenza A H3	11 (36.67)
Rhinovirus	10 (33.33)

**Table 2:** Odds ratios for lipid clusters expressed as crude and adjusted for confounders

OR Crude (95% CI)			
	Clusters		
	1	2	3
Influenza A H1-2009	9.00 (0.56, 143.88)	4.00 (0.45, 35.79)	Ref.
Influenza A H3	15.00 (1.03, 218.29)*	4.00 (0.45, 35.79)	Ref.
Rhinovirus	Ref.	Ref.	–

OR Crude (95% CI)			
	Clusters		
	1	2	3
Influenza A H1-2009	7.96 (0.36, 173.88)	2.36 (0.16, 35.43)	Ref.
Influenza A H3	16.00 (0.59, 433.03)^†^	3.38 (0.19, 60.41)	Ref.
Rhinovirus	Ref.	Ref.	–

*p-value = 0.047

^†^p-value = 0.099

### Identification and classification of sputum lipids by class and species

**Fig. 1:**
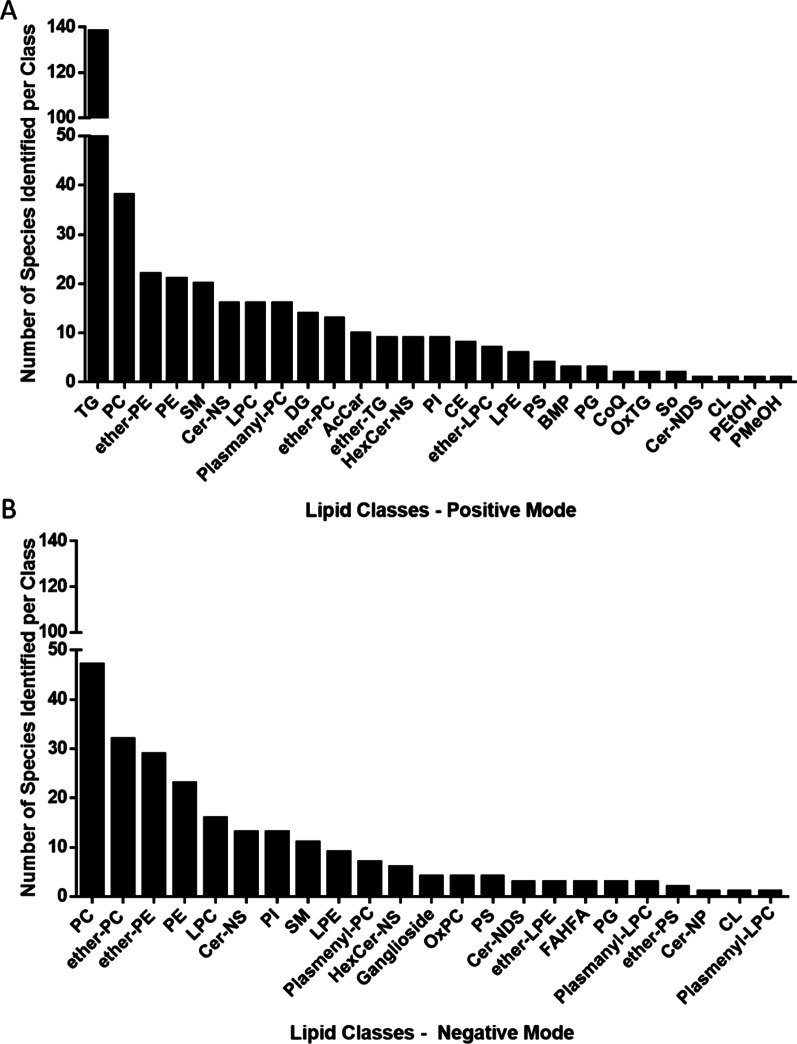
Number of lipid species identified by mass spectrometry across all sputum samples (N = 30) for each lipid class identified in both the **A** positive and **B** negative modes

**Fig. 2:**
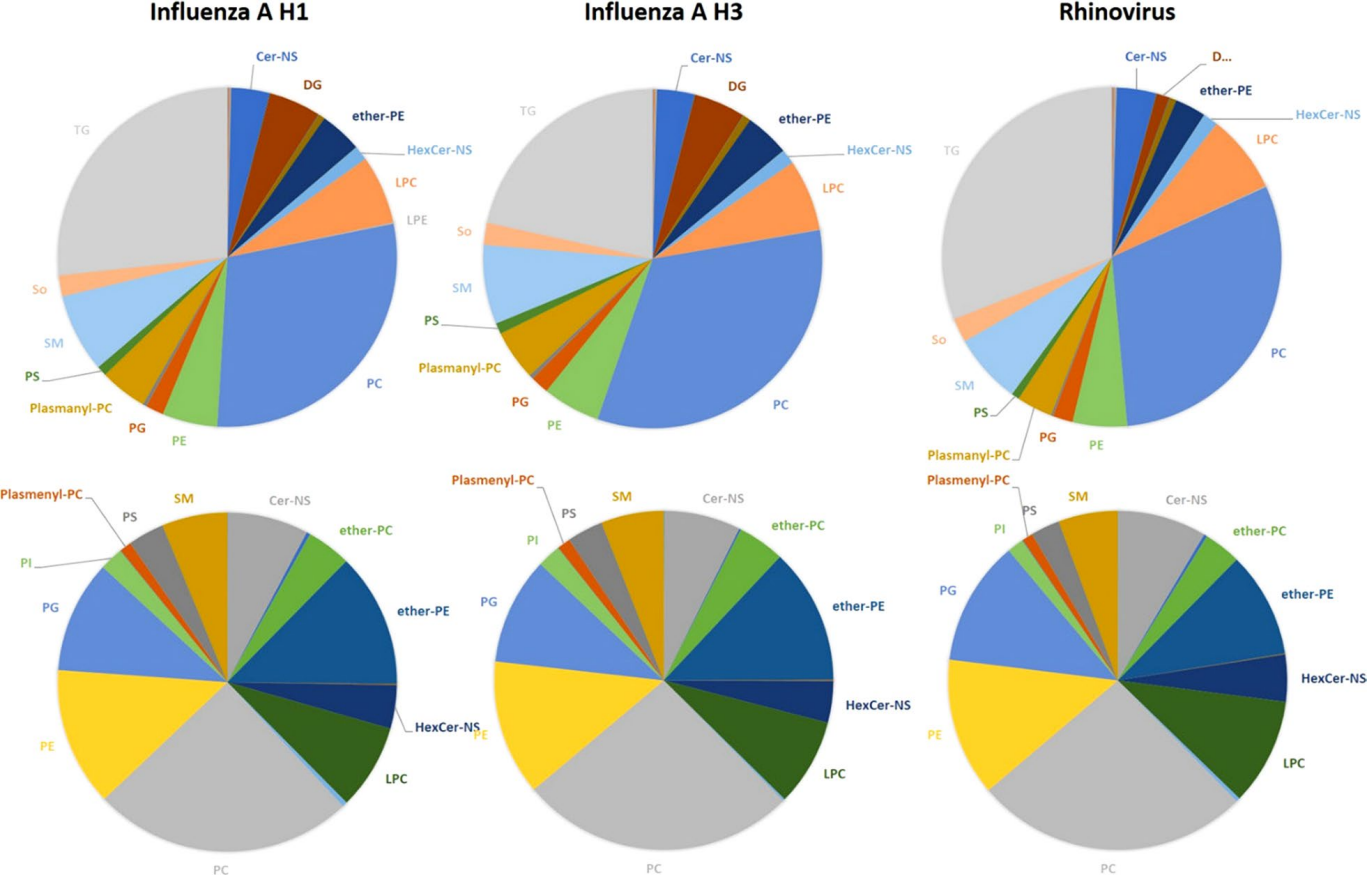
Percent of lipid classes identified by mass spectrometry and stratified by infection type, influenza A H1 (N = 9), Influenza A H3 (N = 11) and Rhinovirus (N = 10), in the positive (upper panel) and negative (lower panel) modes. Note that only lipid classes that make up greater than 5% of the total lipid profile are labeled

**Fig. 3:**
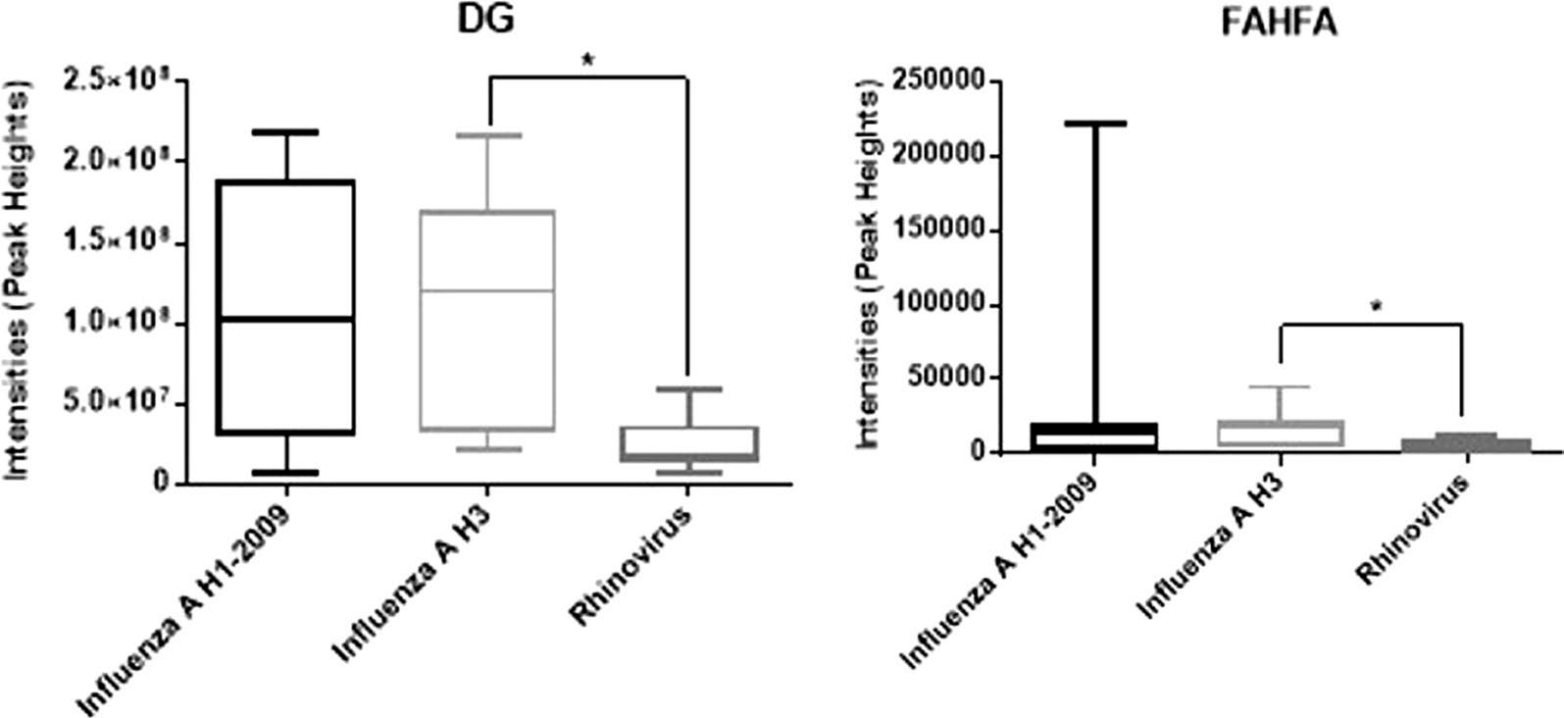
Mean intensity based on peak height of spectra for Diacylglycerols (DG) and Fatty Acid ester of Hydroxyl Fatty Acid (FAHFA) for each infection type, influenza A H1 (N = 9), Influenza A H3 (N = 11) and Rhinovirus (N = 10). Significant differences in intensity were determined by Kruskal–Wallis (P < 0.05) and marked with asterisks

**Fig. 4:**
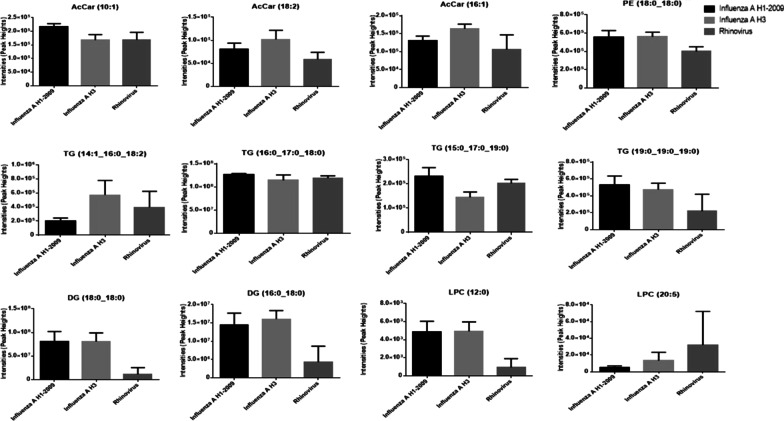
Lipid species that showed significantly different intensity levels in sputum based on infection type for lipids identified in the positive mode, influenza A H1 (N = 9), Influenza A H3 (N = 11) and Rhinovirus (N = 10). All lipids showed significant differences in intensity based on Kruskal–Wallis analysis (p < 0.05). These are a subset of the lipids that were identified in the variable selection step prior to clustering (see “Methods” section for details)

Across all samples, 27 and 23 lipid classes were identified in the positive (Fig. 1A), and negative modes (Fig. 1B), respectively. The most abundant lipid classes identified were TG, PE, PC, SM, ether-PC, and ether-PE. Evaluating the level of lipids at the class level across respiratory infection type yielded profiles that were similar (Fig. 2). Only two lipid classes showed significant differences by infection type, DG and FAHFA (Fig. 3), that were lower in sputum samples from patients that tested positive for Rhinovirus compared to Influenza H3N2. Other lipid classes for which no significant differences were noted are presented in Additional file 1: Figure S2. At the species level, 392 lipid species in the positive mode and 237 lipid species in the negative mode were identified. Kruskal–Wallis tests showed that the levels of 12 lipid species, mostly saturated lipids, were significantly different when stratified by infection type and included AcCar (10:1), AcCar (16:1), AcCar (18:2), DG (16:0_18:0), DG (18:0_18:0), LPC (12:0), LPC (20:5), PE (18:0_18:0), TG (14:1_16:0_18:2), TG (15:0_17:0_19:0), TG (16:0_17:0_18:0), TG (19:0_19:0_19:0) (Fig. 4).

**Fig. 5:**
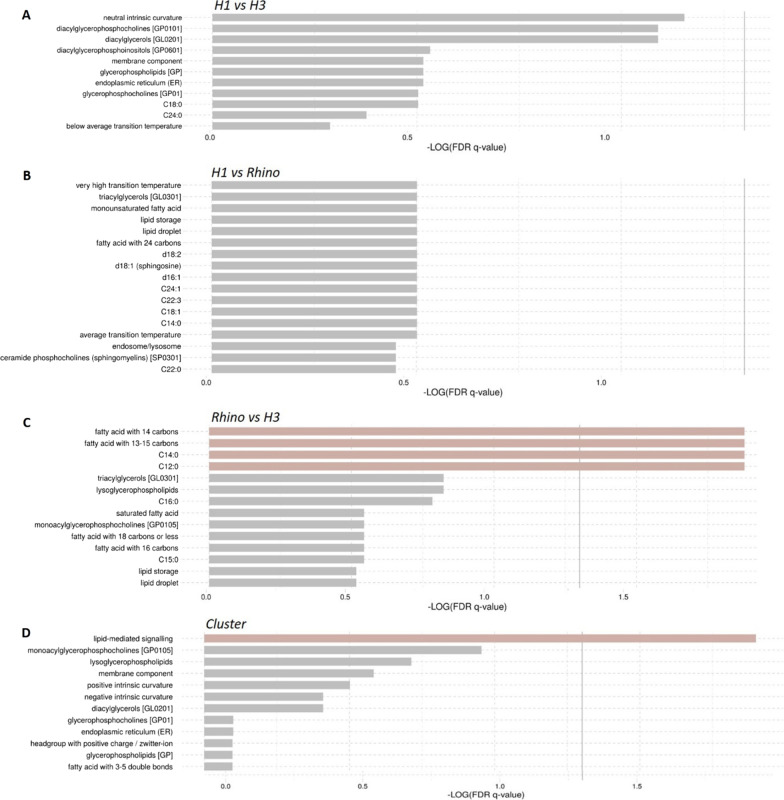
Lipid ontology enrichment analysis (LION) was performed to associate lipids by infection type to chemical and biological features. Using the ranking mode, input lipids are ranked by numeric values and compared between two groups which included; **A** H1 vs H3; **B** H1 vs Rhino; **C** Rhino vs H3. **D** Enrichment analysis was also performed using the target-list mode where the subset of 35 lipids (N = 30) identified as driving the clustering was compared to the total set of lipids Significant enrichments based on *p* < 0.05 are shown for each analysis with the gray vertical lines indicating the cut-off value of significant enrichments based on *q* < 0.05. Bar colors are scaled with the enrichment (− log *q*-values)

Lipid ontology enrichment analysis was performed to associate lipids by infection type to chemical and biological features. Using the ranking mode, input lipids are ranked by numeric values and compared between two groups. Figure 5 shows the enriched pathways/functions for (H1 vs H3, H1 vs Rhino, and Rhino vs H3). In general, the H3 samples were enriched for lipids associated with neutral intrinsic curvature and diacylglycerols (compared to H1); Rhinovirus samples were enriched for processes associated with temperature transitions, triacylglycerols, lipid storage, and lipid droplet formation (compared to H1); H3 showed the most significant association with fatty acids (compared to Rhinovirus).

### Lipid profile cluster and regression analyses

Lipids with a p-value of < 0.2 in a Kruskal–Wallis analysis comparing the lipid expression level for each infection type were selected for Bayesian profile regression clustering (35 lipids in the positive mode, 9 lipids in the negative mode). To get a sense for the functional role of these lipids, lipid ontology enrichment analysis was performed using the target-list mode where this subset of 35 lipids was compared to the total set of lipids. Results show the most significantly enriched pathways are related to glycerolipids, lipids that have headgroups with neutral charges, and triacylglycerols which collectively represent DGs and TGs (Fig. 5D).

**Fig. 6:**
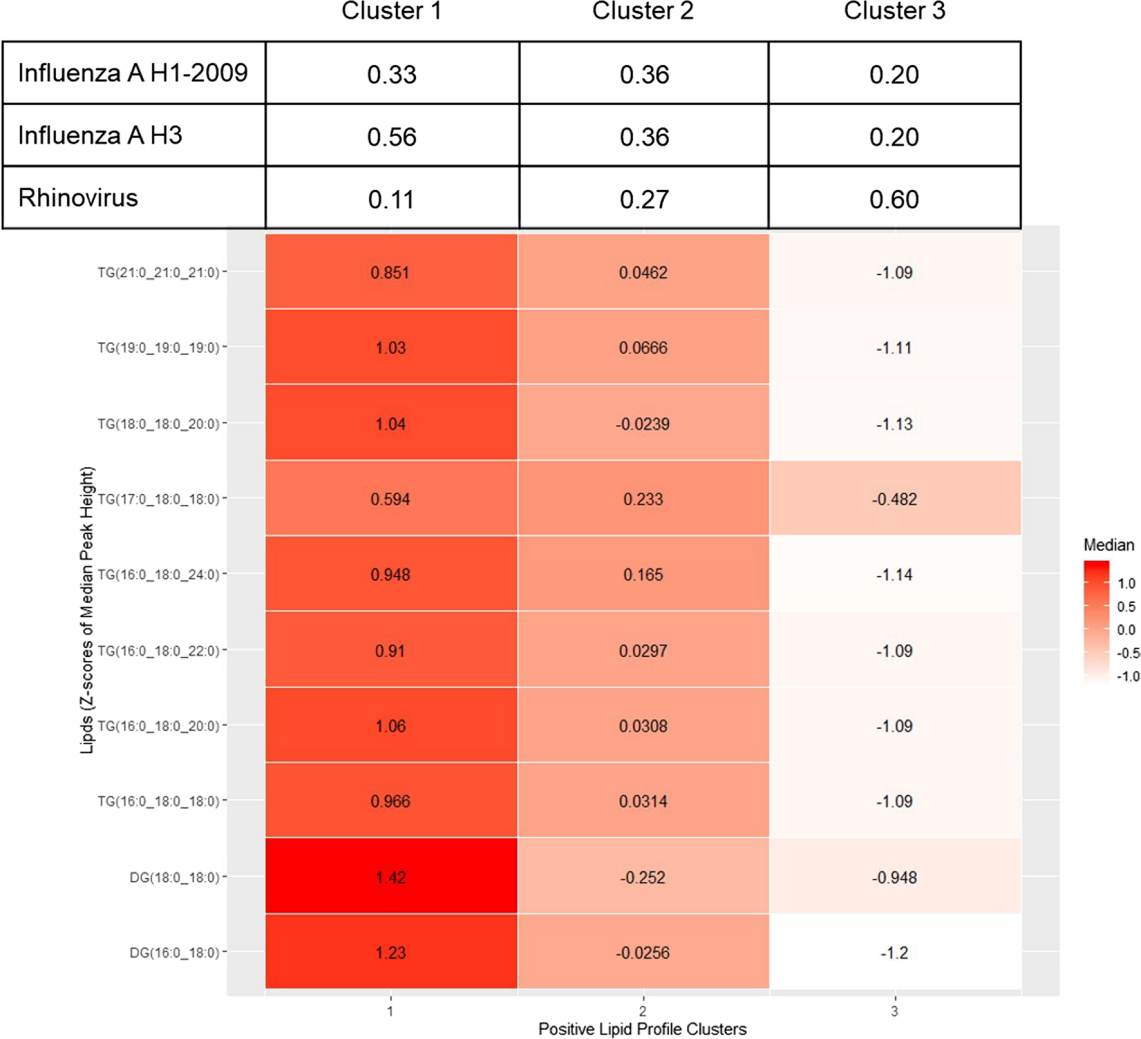
Heat map showing the ten lipid species driving the clusters and their association with infection type *determined by* Bayesian profile regression and multinomial logistic regression. Z-scores for each of the lipid species were calculated from the data (N = 30) and then median values of Z-scores within each cluster were calculated. Darker red colors indicate higher lipid levels and lighter colors represent lower lipid levels for each species for each infection type influenza A H1 (N = 9), Influenza A H3 (N = 11) and Rhinovirus (N = 10)

Cluster analysis (omitting infection type) with Bayesian profile regression was used as a variable selection step prior to multinomial logistic regression, so only those lipid species driving the clustering (median > 0.5) were included in the final regression model. Clustering with variable selection yielded three clusters of lipid profiles in the positive mode and two clusters in the negative mode. In the positive mode, those three clusters were driven by just 10 of the 35 selected lipid species. In the negative mode, only 9 lipids were selected for clustering, and the two yielded clusters were not driven by any specific species, suggesting that the lipids identified in the negative mode are more similar across the patient samples. Because of this, no further analyses were conducted with the negative mode lipids. Heat maps (using Z-scores to put median peak lipid values on the same numeric scale) are presented for each lipid species in each cluster (Fig. 6). In the positive mode, cluster 1 has higher levels of each lipid species and the highest prevalence of influenza A H3 infection (56%, n = 5) (Fig. 6). Cluster 3, overall, has lower levels of each lipid species and the highest prevalence of rhinovirus (60%; n = 6). Cluster 2 has roughly median levels of each lipid species with 36% of the individuals positive for Influenza A H1-2009 (n = 4), 36% positive for Influenza A H3 (n = 4), and 27% positive for Rhinovirus (n = 3). Cluster 3 was set as the reference group for the crude and adjusted multinomial regression models. The crude odds of influenza A H3 infection compared to rhinovirus in cluster 1 (with cluster 3 as reference group) was significantly (p = 0.047) higher (OR = 15.00 [95% CI: 1.03, 218.29]) (Table 2). After adjustment for confounders (smoking status [never, ever, or current], and pulmonary comorbidities), the odds ratio (OR) became only marginally significant (p = 0.099), but the magnitude of the effect estimate was similar (OR = 16.00 [0.59, 433.03]) (Table 2).

## Discussion

In order to investigate associations between sputum lipid profiles and viral respiratory infections, thirty sputum samples from patients with active viral respiratory infections (influenza A H1-2009, influenza A H3, rhinovirus) underwent lipidomics analyses. Statistical analysis was performed to identify differences in the abundance of lipid species between viral infection status, and Bayesian Profile Regression coupled with multinomial logistic regression was then utilized to group similar lipid profiles and examine the association of these profiles with infection type. This is the first study, to our knowledge, that examines potential links between a variety of viral respiratory pathogens and several different classes of lipids, at the species level, in sputum. Another unique strength of this work is the use of both conventional multivariate analyses and a dimension reduction clustering technique (Bayesian Profile Regression) to analyze the lipidomic data.

The lipidomics analyses identified more than 600 unique lipid species in our sputum samples, with TG, PC, and PE as some of the most abundant species. This lipid coverage is similar to sputum profiling reported by Brandsma and colleagues who identified about 100 known species of lipids in sputum, with PC and PE presenting as the most abundant classes [24], and Dushianthan and colleagues who reported PC as the most abundant lipid class in sputum [16]. Work by Telenga, t’Kindt, and colleagues profiled over 1,500 lipids in sputum samples, which is substantially more than our work. However they still identified PC as the most abundant lipid, along with TG [12, 25], which is very similar to the profiles identified here.

Much of the previously published work on lipids in sputum focuses on either healthy populations, smokers, or patients with cystic fibrosis, asthma, or COPD. In our study, we did not have any healthy controls (because we were using sputum samples that are part of routine diagnostic care for those with respiratory illnesses), and we excluded patients with cystic fibrosis. However, we did collect information from medical records regarding smoking status, asthma, and COPD. We observed that about 30% of our population were current smokers (> national average of 15.5% in 2016 [26]) and 43% of our population had asthma, COPD, or both. This rate of pulmonary comorbidities is high compared to other published data which suggests the prevalence of COPD in working adults ages 40 to 70 years is 4.2% [27], and the prevalence of asthma, COPD, or both in those 65 years and older is 9.9%, 9.7%, and 3.0%, respectively [28]. These differences are important to note because there is substantial evidence that smoking, asthma, and COPD can increase a person’s risk of respiratory infections and subsequent complications [29–36]. Additionally, lipidomic studies of sputum or BALF have revealed increased sphingolipid levels in smokers with COPD compared to smokers without COPD and never-smokers [12, 25]; increased levels of lyso-PC (LPC), PC, phosphatidylglycerols (PG), phosphatidylserines (PS), SM, and TG in asthmatics compared to healthy controls [14]; and increased leukotriene and decreased prostaglandin levels in smokers with asthma compared to never smokers with asthma [37].

Using a multifaceted statistical approach, we identified several lipid species that were differentially expressed based on infection type. While we do not fully understand the functional significance of these results, we found that DGs and TGs were strongly upregulated in patients with Influenza H3N2 and clustered together as shown in the heat map in Fig. 7. Of note, multinomial regression showed the odds of influenza A H3 infection relative to odds of rhinovirus were 15 times higher when comparing cluster 1 and cluster 3 (OR = 15.00 [1.03, 218.29]). Interestingly, TG content has been shown to be critical to lipid droplet formation, a process that may be beneficial to viral replication, but may also serve to enhance the host immune system [38]. These results suggest that a profile of increased TG and DG lipids could be associated with influenza A H3 infection and are excellent candidates for future research.

Others have reported higher lipid levels concurrent with infection that are similar to what is observed in this population. For example, To and colleagues observed higher SM, Hex-Cer, and LPC species and lower lyso-PE (LPE) species in community-acquired compared to non-community acquired pneumonia patients [13]. Although we did not observe increases in the exact same species, we did detect changes in species from those classes (i.e. LPC), which were selected during our initial variable selection step. It is interesting that LPC 12:0 is higher in sputum from patients with influenza (H1N1 and H3N2) whereas the greatest levels of LPC 20:5 occurred in individuals infected with RV. LPC has been shown to enhance inflammatory pathways through production of arachidonic acid and downstream eicosanoids or by inhibition of viral fusion with host cell membranes [39]. LPC(20:0) has been positively associated with patrolling monocytes in patients with HIV [40] but the exact role of these particular LPC species in the host response to respiratory viruses remains to be determined. Other studies have reported significant increases in SM in the BALF of influenza-infected mice [41] and fatty acid elongation and desaturation during rhinovirus infection of human bronchial epithelial cells [42]. They suggested that these changes in FA metabolism play a role in supporting Rhinovirus replication since DG can activate protein kinase D, the inhibition of which has been previously shown to reduce rhinovirus replication [42]. In our study, we observed lower levels for several TG species that drove the clustering pattern, including those with up to 24 carbons in their acyl chains. Our results, coupled with other reports from the literature, suggest that these links between changes in lipid levels and respiratory infections may have mechanistic validity and thus potentially diagnostic, prognostic, or therapeutic implications.

We acknowledge that this study is not without limitations. First and foremost, it was a small study with fairly low sample size and did not include a control group (i.e. those known to be without a respiratory infection or high sputum conditions without respiratory infection). However, despite the small sample size, we were able to logically compare our results to other studies where lipids from control individuals were reported. Importantly, we were also able to observe significant associations, which emphasizes the need to continue this line of research with a larger sample size and control group in order to better characterize and substantiate these interesting and novel findings. Because this is a cross-sectional study, there was also no way to determine the temporality of these lipid changes from onset to progression of infection. Therefore, we acknowledge it is equally possible that the patients had perturbed lipid profiles prior to infection and were therefore more susceptible to infection or that the lipid profiles were perturbed as a result of the infection. Prospective cohort studies are needed to tease out the temporal component of these lipid changes. Finally, we are aware that the sputum likely contained leukocytes which contributed to the overall lipid profiles generated. The contribution of leukocytes is important as the sputum of individuals with health conditions such as infections will commonly contain leukocytes. Despite these limitations, this cross-sectional study was successful in examining possible associations between lipid profiles of sputum and viral respiratory infections that have both mechanistic and clinical importance.

## Conclusions

This study identified a significant association for a novel cluster of lipid profiles from the positive ionization mode, and the odds of influenza A H3 infection compared to rhinovirus were 15 times higher when comparing cluster 1 and cluster 3 (OR = 15.00 [1.03, 218.29]). Despite the lack of statistical significance after adjustment, this large effect size, the magnitude of which did not substantially change after adjustment, supports our hypothesis that different lipid profiles are associated with different types of viral infection, and strongly endorses the continuation of this work with larger sample sizes in order to better characterize these novel findings. Work such as this can contribute to the growing body of literature on the role of lipids in respiratory infection pathogenesis, perhaps as a molecular initiating event that results in increased susceptibility or for potential use as therapeutics or biomarkers.

## Supplementary Information

**Additional file 1: Table S1.** Optimization of the BioFire FilmArray® platform for sputum samples. **Table S2.** Concordance of pathogen identification between nasalpharyngeal (NP) swab and sputum samples. **Table S3.** Number of lipids identified in each class and percent of total lipids for positive and negative mode. **Figure S1.** Causal diagram, also known as directed acyclic graph (DAG), that presents a visualization of the other variables that may play a role in the relationship between lung lipids (exposure) and viral respiratory infection (outcome). **Figure S2.** Intensity of all lipids identified in the positive (S1A) and negative (S1B) modes.

## Data Availability

The datasets used and/or analyzed during the current study are available from the corresponding author on reasonable request.

## Abbreviations

CDC: Centers for Disease Control and Prevention
WHO: World Health Organization
Cer: Ceramides
SM: Sphingomyelins
PE: Phosphatidylethanolamines
PC: Phosphatidylcholines
BALF: Bronchoalveolar lavage fluid
COPD: Chronic obstructive pulmonary disease
UF: University of Florida
DTT: Dithiothreitol
SECIM: Southeast Center for Integrated Metabolomics
RCP: Red Cross Plasma
PEG: Polyethylene glycol
BMI: Body mass index
DAG: Directed acyclic graph
DG: Diacylglycerol
FAHFA: Fatty Acid ester of Hydroxyl Fatty Acid
AcCar: Acylcarnitine
LPC: Lysophosphatidylcholine
TG: Triglycerides
OR: Odds ratio
PG: Phosphatidylglycerols
PS: Phosphatidylserines

## References

[1] WHO | The top 10 causes of death. WHO/entity/mediacentre/factsheets/fs310/en/index.html.

[2] Arbex MA, Santiago SL, Moyses EP, Pereira LA, Saldiva PH, Braga ALF. Impact of Urban Air Pollution on Acute Upper Respiratory Tract Infections. In: Moldoveanu A, editor. *Adv Top Environ Health Air Pollut Case Stud* InTech; 2011. http://www.intechopen.com/books/advanced-topicsin- environmental-health-and-air-pollution-case-studies/impact-of-urban-air-pollution-on-acute-upperrespiratory- tract-infections.

[3] Disease Burden of Influenza | Seasonal Influenza (Flu) | CDC. 2017. https://www.cdc.gov/flu/about/disease/burden.htm.

[4] World Health Organization. WHO Public Health Research Agenda for Influenza. 2017.

[5] Köberlin MS, Snijder B, Heinz LX, Baumann CL, Fauster A, Vladimer GI, Gavin A-C, Superti-Furga G (2015). A conserved circular network of coregulated lipids modulates innate immune responses. Cell.

[6] Tisoncik-Go J, Gasper DJ, Kyle JE, Eisfeld AJ, Selinger C, Hatta M, Morrison J, Korth MJ, Zink EM, Kim Y-M, Schepmoes AA, Nicora CD, Purvine SO, Weitz KK, Peng X, Green RR, Tilton SC, Webb-Robertson B-J, Waters KM, Metz TO, Smith RD, Kawaoka Y, Suresh M, Josset L, Katze MG (2016). Integrated omics analysis of pathogenic host responses during pandemic h1n1 influenza virus infection: the crucial role of lipid metabolism. Cell Host Microbe.

[7] Tanner LB, Chng C, Guan XL, Lei Z, Rozen SG, Wenk MR (2014). Lipidomics identifies a requirement for peroxisomal function during influenza virus replication. J Lipid Res.

[8] Morita M, Kuba K, Ichikawa A, Nakayama M, Katahira J, Iwamoto R, Watanebe T, Sakabe S, Daidoji T, Nakamura S, Kadowaki A, Ohto T, Nakanishi H, Taguchi R, Nakaya T, Murakami M, Yoneda Y, Arai H, Kawaoka Y, Penninger JM, Arita M, Imai Y (2013). The lipid mediator protectin d1 inhibits influenza virus replication and improves severe influenza. Cell.

[9] Tam VC (2013). Lipidomic profiling of bioactive lipids by mass spectrometry during microbial infections. Semin Immunol.

[10] Köberlin MS, Heinz LX, Superti-Furga G (2016). Functional crosstalk between membrane lipids and TLR biology. Curr Opin Cell Biol.

[11] Zhao Y-Y, Cheng X-L, Lin R-C, Wei F (2015). Lipidomics applications for disease biomarker discovery in mammal models. Biomark Med.

[12] Kindt R, Telenga ED, Jorge L, Van Oosterhout AJM, Sandra P, TenHacken NHT, Sandra K (2015). Profiling over 1500 Lipids in Induced Lung Sputum and the Implications in Studying Lung Diseases. Anal Chem.

[13] To KKW, Lee K-C, Wong SSY, Sze K-H, Ke Y-H, Lui Y-M, Tang BSF, Li IWS, Lau SKP, Hung IFN, Law C-Y, Lam C-W, Yuen K-Y (2016). Lipid metabolites as potential diagnostic and prognostic biomarkers for acute community acquired pneumonia. Diagn Microbiol Infect Dis.

[14] Kang YP, Lee WJ, Hong JY, Lee SB, Park JH, Kim D, Park S, Park C-S, Park S-W, Kwon SW (2014). Novel Approach for Analysis of Bronchoalveolar Lavage Fluid (BALF) Using HPLC-QTOF-MS-based lipidomics: lipid levels in asthmatics and corticosteroid-treated asthmatic patients. J Proteome Res.

[15] Wheelock CE, Goss VM, Balgoma D, Nicholas B, Brandsma J, Skipp PJ, Snowden S, Burg D, D’Amico A, Horvath I, Chaiboonchoe A, Ahmed H, Ballereau S, Rossios C, Chung KF, Montuschi P, Fowler SJ, Adcock IM, Postle AD, Dahlén S-E, Rowe A, Sterk PJ, Auffray C, Djukanović R (2013). Application of ’omics technologies to biomarker discovery in inflammatory lung diseases. Eur Respir J.

[16] Dushianthan A, Goss V, Cusack R, Grocott MP, Postle AD (2014). Phospholipid composition and kinetics in different endobronchial fractions from healthy volunteers. BMC Pulm Med.

[17] Cho M-C, Kim H, An D, Lee M, Noh S-A, Kim M-N, Chong YP, Woo JH (2012). Comparison of Sputum and Nasopharyngeal Swab Specimens for Molecular Diagnosis of Mycoplasma pneumoniae, Chlamydophila pneumoniae, and Legionella pneumophila. Ann Lab Med.

[18] Jeong JH, Kim KH, Jeong SH, Park JW, Lee SM, Seo YH (2014). Comparison of sputum and nasopharyngeal swabs for detection of respiratory viruses. J Med Virol.

[19] Covalciuc KA, Webb KH, Carlson CA (1999). Comparison of four clinical specimen types for detection of influenza A and B viruses by optical immunoassay (FLU OIA test) and cell culture methods. J Clin Microbiol.

[20] Folch J, Lees M, Stanley GS (1957). A simple method for the isolation and purification of total lipides from animal tissues. J Biol Chem.

[21] Koelmel JP, Kroeger NM, Ulmer CZ, Bowden JA, Patterson RE, Cochran JA, Beecher CWW, Garrett TJ, Yost RA (2017). LipidMatch: an automated workflow for rule-based lipid identification using untargeted high-resolution tandem mass spectrometry data. BMC Bioinformatics.

[22] Coker E, Gunier R, Bradman A, Harley K, Kogut K, Molitor J, Eskenazi B (2017). Association between pesticide profiles used on agricultural fields near maternal residences during pregnancy and IQ at age 7 years. Int J Environ Res Public Health.

[23] Coker E, Liverani S, Su JG, Molitor J (2018). Multi-pollutant modeling through examination of susceptible subpopulations using profile regression. Curr Environ Health Rep.

[24] Brandsma J, Goss VM, Yang X, Bakke PS, Caruso M, Chanez P, Dahlén S-E, Fowler SJ, Horvath I, Krug N (2018). Lipid phenotyping of lung epithelial lining fluid in healthy human volunteers. Metabolomics.

[25] Telenga ED, Hoffmann RF, Kindt R, Hoonhorst SJ, Willemse BW, van Oosterhout AJ, Heijink IH, van Berge M, Jorge L, Sandra P (2014). Untargeted lipidomic analysis in chronic obstructive pulmonary disease Uncovering sphingolipids. Am J Respir Crit Care Med.

[26] Jamal A, Phillips E, Gentzke AS, Homa DM, Babb SD, King BA, Neff LJ (2018). Current cigarette smoking among adults—United States, 2016. Morb Mortal Wkly Rep.

[27] Doney B, Hnizdo E, Syamlal G, Kullman G, Burchfiel C, Martin CJ, Mujuru P (2014). Prevalence of chronic obstructive pulmonary disease among US working adults aged 40 to 70 years: National Health Interview Survey Data 2004 to 2011. J Occup Environ Med Coll Occup Environ Med.

[28] Oraka E, Kim HJE, King ME, Callahan DB (2012). Asthma prevalence among US elderly by age groups: age still matters. J Asthma.

[29] Bouneb R, Mellouli M, Bensoltane H, Baroudi J, Chouchene I, Boussarsar M (2018). Characteristics and outcome of ill critical patients with influenza A infection. Pan Afr Med J.

[30] Gorse GJ, Donovan MM, Patel GB, Balasubramanian S, Lusk RH (2015). Coronavirus and other respiratory illnesses comparing older with young adults. Am J Med.

[31] Greenberg SB (2002). Viral respiratory infections in elderly patients and patients with chronic obstructive pulmonary disease. Am J Med.

[32] Monto AS, Fendrick AM, Sarnes MW (2001). Respiratory illness caused by picornavirus infection: a review of clinical outcomes. Clin Ther.

[33] Kalil AC, Thomas PG (2019). Influenza virus-related critical illness: pathophysiology and epidemiology. Crit Care.

[34] Kwak HJ, Park DW, Kim JE, Park MK, Koo GW, Park TS, Moon J-Y, Kim TH, Sohn JW, Yoon HJ (2016). Prevalence and risk factors of respiratory viral infections in exacerbations of chronic obstructive pulmonary disease. Tohoku J Exp Med.

[35] Miller EK, Linder J, Kraft D, Johnson M, Lu P, Saville BR, Williams JV, Griffin MR, Talbot HK (2016). Hospitalizations and Outpatient Visits for Rhinovirus-Associated Acute Respiratory Illness in Adults. J Allergy Clin Immunol.

[36] Nicholson KG, Kent J, Hammersley V, Cancio E (1996). Risk factors for lower respiratory complications of rhinovirus infections in elderly people living in the community: prospective cohort study. BMJ.

[37] Thomson NC, Chaudhuri R, Spears M, Messow CM, Jelinsky S, Miele G, Nocka K, Takahashi E, Hilmi OJ, Shepherd MC, Miller DK, McSharry C (2014). Arachidonic acid metabolites and enzyme transcripts in asthma are altered by cigarette smoking. Allergy.

[38] Monson EA, Trenerry AM, Laws JL, Mackenzie JM, Helbig KJ (2021). Lipid droplets and lipid mediators in viral infection and immunity. FEMS Microbiol Rev.

[39] Günther-Ausborn S, Praetor A, Stegmann T (1995). Inhibition of Influenza-induced Membrane Fusion by Lysophosphatidylcholine. J Biol Chem.

[40] Bowman ER, Kulkarni M, Gabriel J, Cichon MJ, Riedl K, Belury MA, Lake JE, Richardson B, Cameron C, Cameron M, Koletar SL, Lederman MM, Sieg SF, Funderburg NT (2019). Altered Lipidome Composition Is Related to Markers of Monocyte and Immune Activation in Antiretroviral Therapy Treated Human Immunodeficiency Virus (HIV) Infection and in Uninfected Persons. Front Immunol.

[41] Woods PS, Doolittle LM, Rosas LE, Joseph LM, Calomeni EP, Davis IC (2016). Lethal H1N1 influenza A virus infection alters the murine alveolar type II cell surfactant lipidome. Am J Physiol-Lung Cell Mol Physiol.

[42] Nguyen A, Guedán A, Mousnier A, Swieboda D, Zhang Q, Horkai D, Le Novere N, Solari R, Wakelam MJ (2018). Host lipidome analysis during rhinovirus replication in HBECs identifies potential therapeutic targets. J Lipid Res.

