# Supplementary material for: Association between lipid profiles and viral respiratory infections in human sputum samples
Source: Respir Res. 2022 Jul 2;23:177. doi: 10.1186/s12931-022-02091-w (PMC9250719; doi:10.1186/s12931-022-02091-w)

Additional Information

Optimization for Pathogen Identification in Sputum

The BioFire FilmArray® Classic Respiratory Panel was utilized for pathogen detection and allows for rapid, high-throughput detection of up to twenty viral and bacterial respiratory pathogens. However, this assay has not been used extensively for sputum samples. For optimization of this assay with sputum samples, we investigated the use of dithiothreitol (DTT) as a mucolytic agent, and with this approach observed that the assay is capable of detecting a spiked amount of influenza A successfully (i.e. no interference by DTT, **Table S1**). These data demonstrate our feasibility to detect respiratory pathogens in sputum samples using the BioFire FilmArray® platform.

Of the thirty sputum samples collected, a subset of them (seven) had sufficient volume for both lipidomics analysis and testing using the BioFire FilmArray® 1.5 Classic Respiratory Panel. Because the infection type was known for all patients based on the results from the Clinical Laboratory, these seven samples were used to examine concordance of the BioFire FilmArray® results when different sample types are used (nasopharyngeal (NP) swab in the Clinical Laboratory compared to sputum in our study). All seven sputum samples tested yielded the same results as the same patient’s NP swab for 100% concordance between the two sample types (**Table S2**).

**Levels of lipids by class in sputum for individuals positive for select viral agents**

The number of lipids total and percent representation of each class are presented in Table S3. Data showing the changes in lipid levels by class that were measured in sputum samples as described in the main text. Lipids identified in the positive mode (S1A) and negative modes (S1B) are graphed as peak height intensities. Note the only significant differences across viral type occurred for DG and FAHFA as shown in Figure 3 in the manuscript.

**Generation of a directed acyclic graph (DAG)**

A DAG (DAGitty version 2.3) was constructed to visualize and identify the confounding variables that may be influencing the relationship between lung lipids (exposure) and viral respiratory infection (outcome). The graph indicates that age, BMI, smoking status, and presence of pulmonary comorbidities such as asthma or COPD could act as possible confounders in our study. We then included only those covariates for adjustment in multinomial regression.

Table S1. Optimization of the BioFire FilmArray® platform for sputum samples


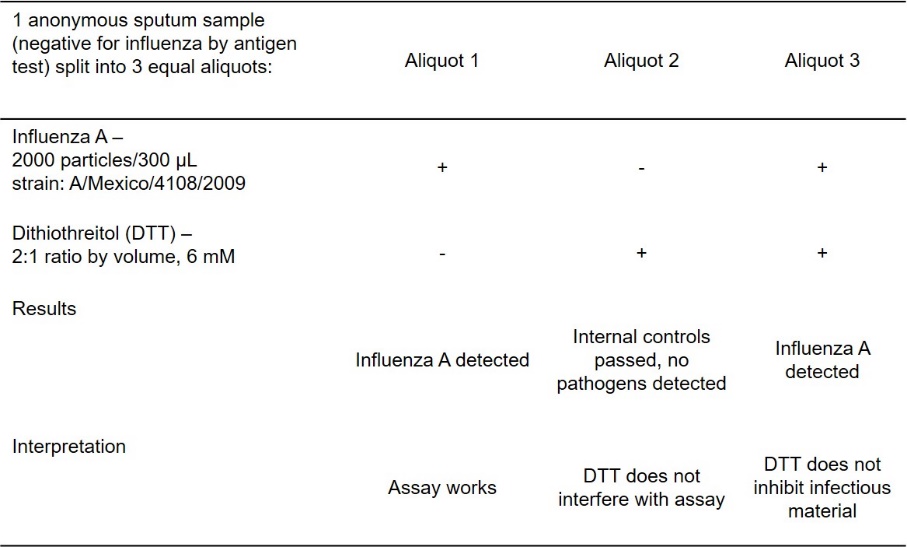


Table S2. Concordance of pathogen identification between nasalpharyngeal (NP) swab and sputum samples


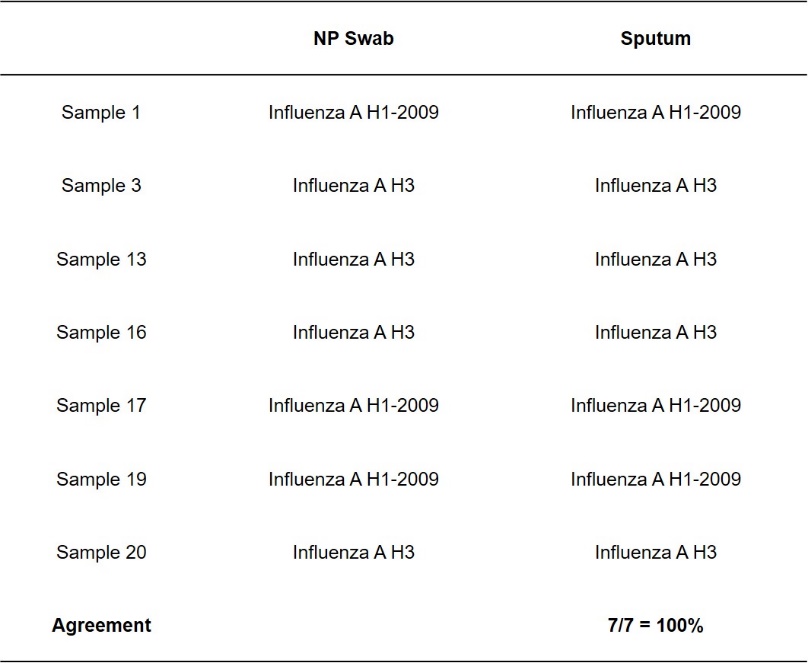


Table S3. Number of lipids identified in each class and percent of total lipids for positive and negative mode


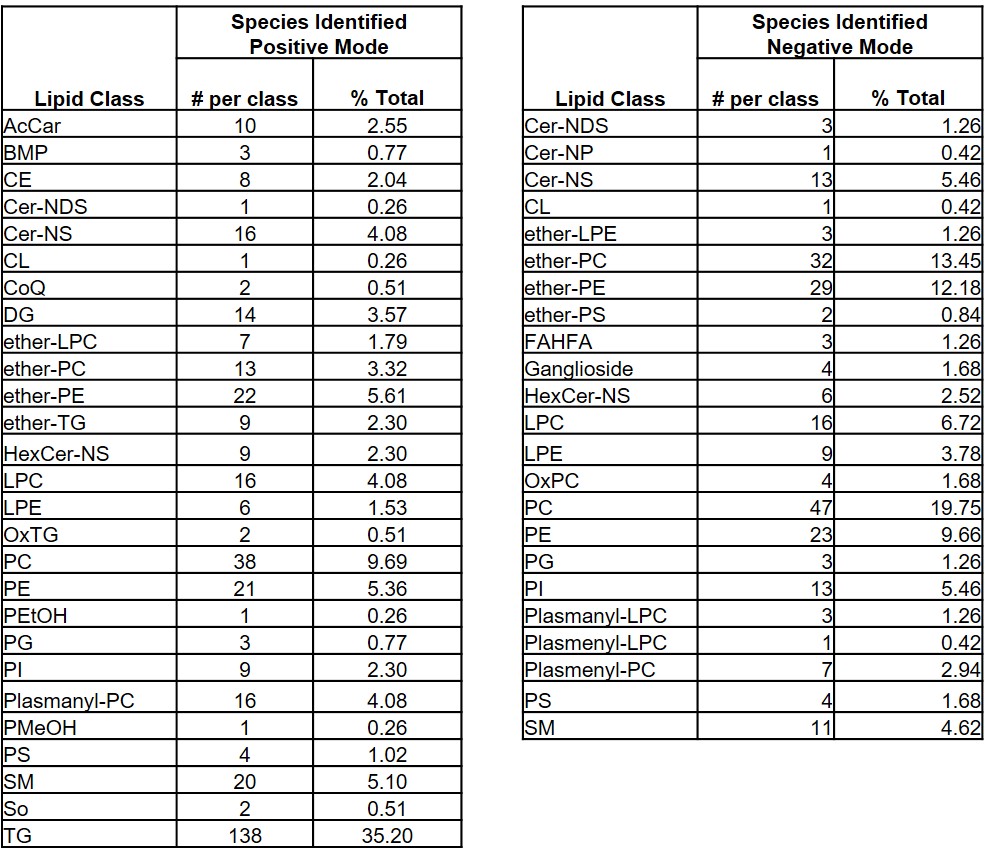


Figure legend S1. Causal diagram, also known as directed acyclic graph (DAG), that presents a visualization of the other variables that may play a role in the relationship between lung lipids (exposure) and viral respiratory infection (outcome).


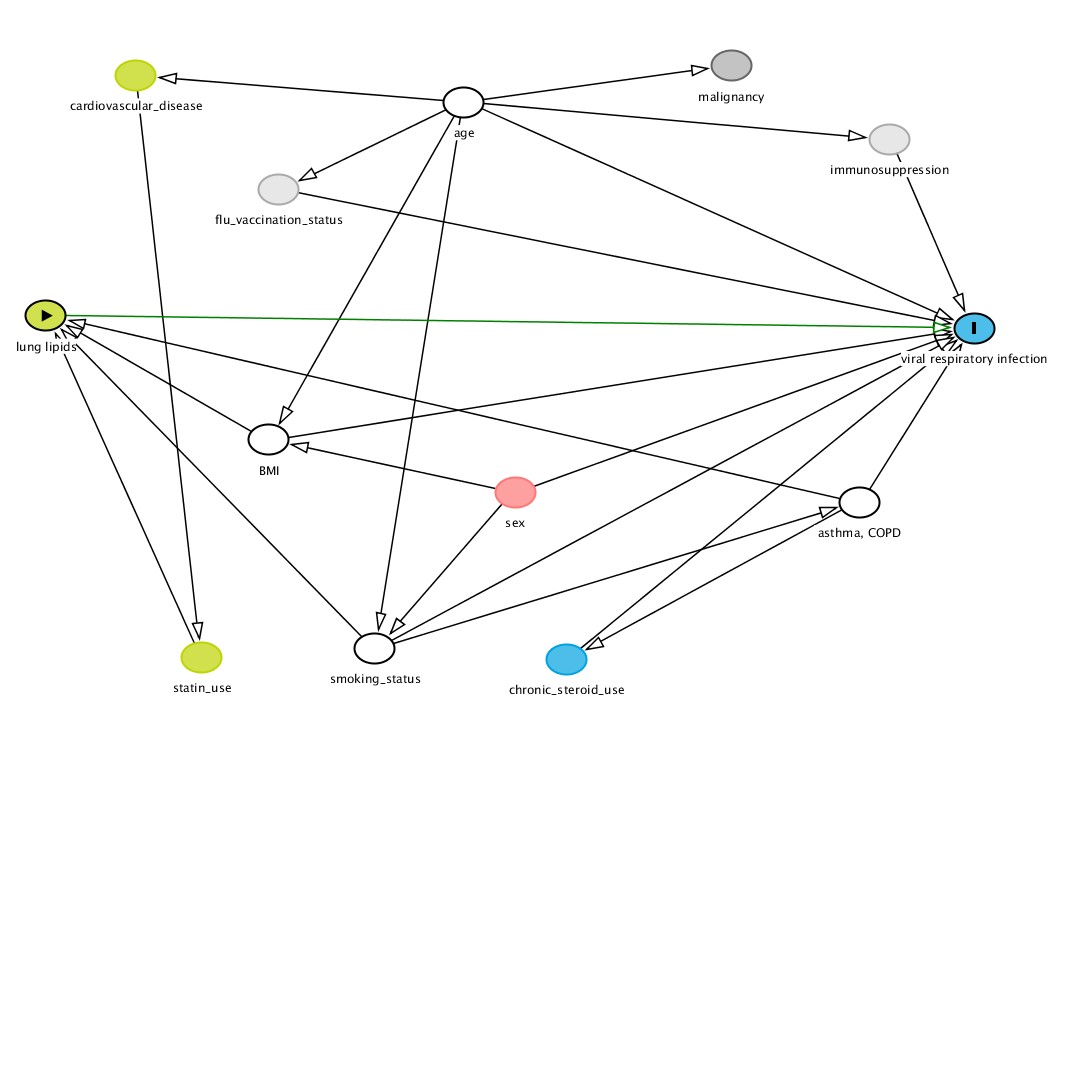


Figure legend S2. Intensity of all lipids identified in the positive (S1A) and negative (S1B) modes.

Figure S2A


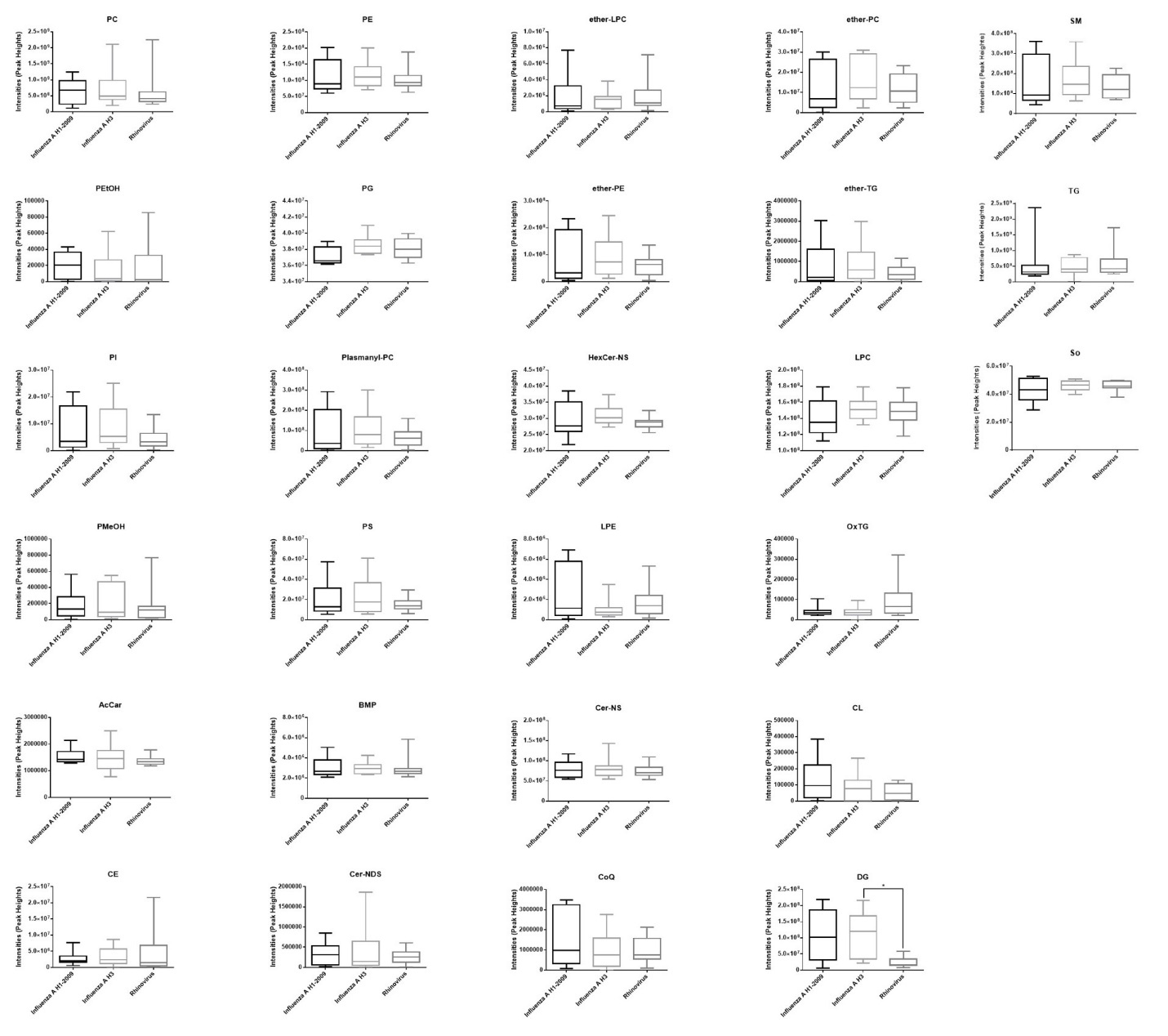


Figure S2B


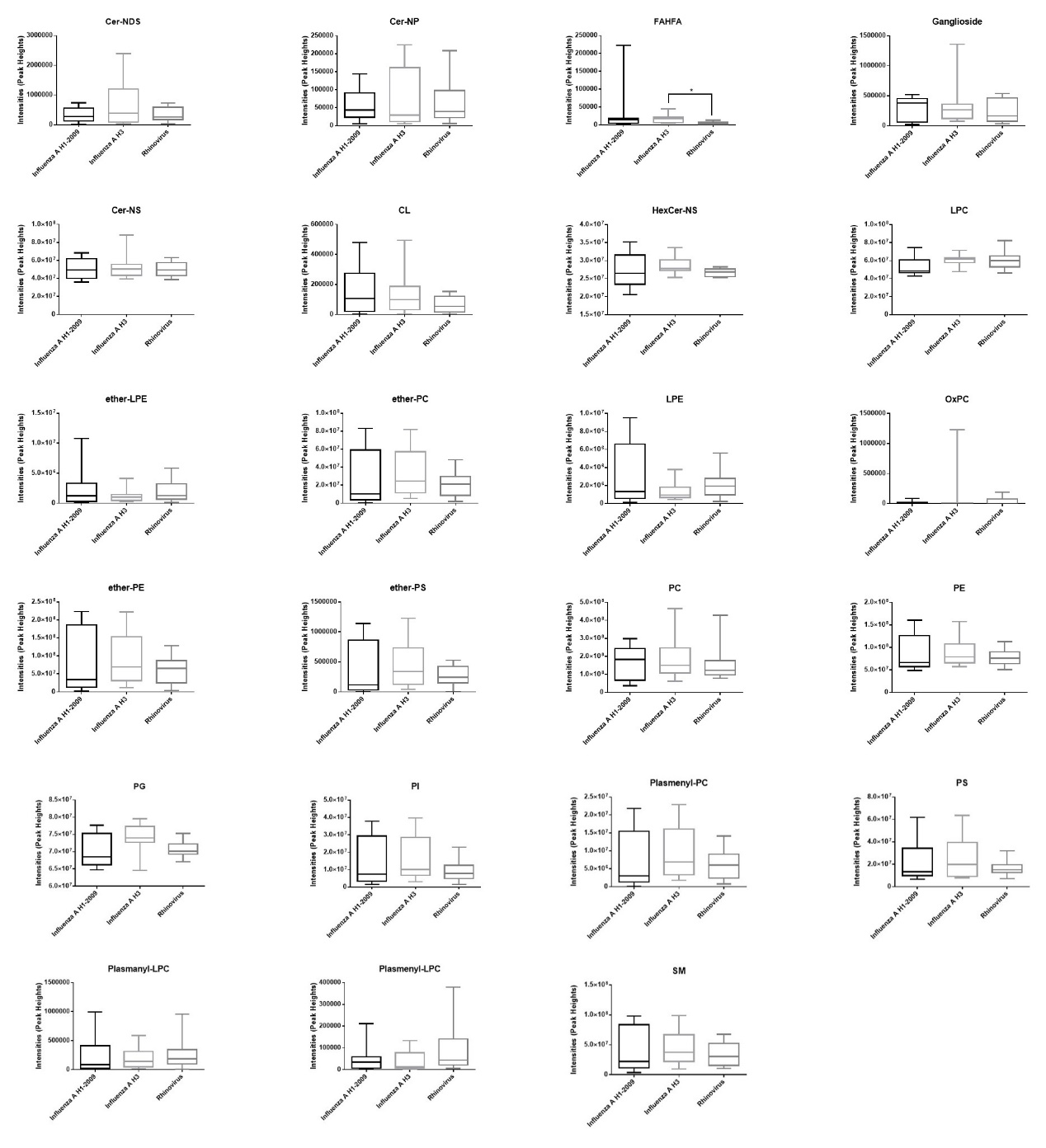

Supplement: Supplementary file 1 — Additional file 1: Table S1. Optimization of the BioFire FilmArray® platform for sputum samples. Table S2. Concordance of pathogen identification between nasalpharyngeal (NP) swab and sputum samples. Table S3. Number of lipids identified in each class and percent of total lipids for positive and negative mode. Figure S1. Causal diagram, also known as directed acyclic graph (DAG), that presents a visualization of the other variables that may play a role in the relationship between lung lipids (exposure) and viral respiratory infection (outcome). Figure S2. Intensity of all lipids identified in the positive (S1A) and negative (S1B) modes. [file 12931_2022_2091_MOESM1_ESM.docx]
